# Facial Expression Training Optimises Viewing Strategy in Children and Adults

**DOI:** 10.1371/journal.pone.0105418

**Published:** 2014-08-21

**Authors:** Petra M. J. Pollux, Sophie Hall, Kun Guo

**Affiliations:** 1 School of Psychology, University of Lincoln, Lincoln, Lincolnshire, United Kingdom; 2 School of Life Science, University of Lincoln, Lincoln, Lincolnshire, United Kingdom; University of British Columbia, Canada

## Abstract

This study investigated whether training-related improvements in facial expression categorization are facilitated by spontaneous changes in gaze behaviour in adults and nine-year old children. Four sessions of a self-paced, free-viewing training task required participants to categorize happy, sad and fear expressions with varying intensities. No instructions about eye movements were given. Eye-movements were recorded in the first and fourth training session. New faces were introduced in session four to establish transfer-effects of learning. Adults focused most on the eyes in all sessions and increased expression categorization accuracy after training coincided with a strengthening of this eye-bias in gaze allocation. In children, training-related behavioural improvements coincided with an overall shift in gaze-focus towards the eyes (resulting in more adult-like gaze-distributions) and towards the mouth for happy faces in the second fixation. Gaze-distributions were not influenced by the expression intensity or by the introduction of new faces. It was proposed that training enhanced the use of a uniform, predominantly eyes-biased, gaze strategy in children in order to optimise extraction of relevant cues for discrimination between subtle facial expressions.

## Introduction

Humans rely on emotional expressions of others to interpret social situations and to flexibly adjust behaviour to the social environment. In accordance, the accurate understanding of facial expressions has been shown to predict better social adjustment, mental health, and even workplace performance [1], [2]. The ability to recognize facial expressions improves greatly with age in childhood [3]–[5] and continues to develop in adolescence for more complex and subtle expressions [5]–[6]. This improvement has been attributed to the development of relevant cognitive and perceptual capacities, as well as increasing practice and exposure over time [4], [5], [7], [8]. Several findings have evidenced a positive association between academic performance in children and nonverbal sensitivity, particularly for facial expressions [2], [9], [10]. For example, Izard et al [10] showed that better skills in facial expression recognition in 5-year-old children positively predicted social and academic outcomes four years later.

Despite the importance of skilled facial expression recognition for social functioning, literature on effective training programs for facial expression categorisation is relatively sparse. Some studies have demonstrated successful benefits of practice (within one session) and training (across several sessions) in facial expression categorization on emotion recognition [11]–[13] and a few findings have demonstrated an association with subsequent improved social functioning [12], [14]. Grispan et al [12] showed for example that six 30-minute training sessions for school children in discrimination, identification and expression of facial expression cues not only improved emotion recognition, it also reduced social anxiety and increased feelings of self-worth, particularly for girls. More recently, Penton-Voak et al [14] demonstrated that the interpretation of ambiguous facial expressions (using morphed facial stimuli with 50% happy and 50% angry expressions) can be biased towards perception of happiness by manipulating feedback in a training task, and that this change in perception is associated with improved social functioning. It was found that training on this task reduced self-reported ratings of anger and aggression in healthy young adults and aggressive behaviour in a group of adolescence considered to be high risk for committing crimes [14].

The processes that underlie improved performance in facial expression recognition training are not yet fully understood. Focusing gaze on internal facial features that provide crucial cues for expression recognition (i.e. eyes, nose and mouth) has been shown to be important, as evidenced by the beneficial effect of gaze instruction in people with impaired facial expression recognition skills [15]–[17]. Whilst this finding highlights the importance of changing gaze, the nature of the relationship between gaze-patterns and behavioural improvements is not yet clear. If the enhancement of facial expression recognition is facilitated by changes in gaze-patterns, then it follows that increased performance (e.g., in normal development, or after training without explicit gaze instructions) should coincide with spontaneous changes in gaze-patterns when viewing faces. Here we aim to investigate this assumption by training adults and children on a facial expression recognition task without gaze instructions and by recording eye-movements in the first and last training session to establish changes in gaze-patterns. Children were included to investigate whether the relationship between behavioural improvement and changes in gaze-patterns is more pronounced when expression recognition skills are still developing. In addition, to investigate if training effects transfer to new faces after training, faces of new models were added to the ‘trained’ faces in the last session.

The training procedure used in the present study involves repeated exposure to different emotional faces that vary in expression intensity in a self-paced free-viewing task requiring categorization responses. Several eye-movement studies have shown that adults generally focus more on the eyes when free-viewing faces for facial expression categorization [18]–[19], whereas a few studies found evidence for emotion-specific gaze-distributions, reflected in enhanced viewing of the eyes for sad or angry faces and more viewing of the mouth for happy faces [20]. Given the evidenced beneficial effect of gaze-instruction on impaired facial expression recognition skills [15]–[17], it could be predicted that behavioural improvements will coincide with a stronger focus on diagnostic facial features for different expressions after training [20]. An alternative prediction can be inferred from findings reported by Guo [19], who used a free-viewing, self-paced facial expression recognition task with emotional faces that varied in expression intensity (from 10% to 100% intensity). Guo [19] found that participants looked most often and for longest at the eyes for all facial expressions with only small variations across different expressions in the distribution of fixations across key facial features. Interestingly, this distribution of eye-movements was unaffected by expression intensity, despite the variation in cue-strength of facial features at low and high expression intensity levels [19]. It was argued that this uniformity in fixation distributions reflects the use of a holistic viewing strategy in order to optimise the extraction of expressive cues from all facial features when discrimination between subtle facial expressions is required [19]. If this viewing strategy is indeed adopted to optimize facial expression recognition, then behavioural benefits of training should coincide with enhanced use of this strategy. This should be reflected in a stronger uniformity across different facial expressions and expression intensity levels in the proportional distribution of fixation and viewing time over the eyes, nose and mouth after training.

Very few studies have investigated children's eye-movement patterns in free-viewing facial expression recognition tasks. A recent Japanese study showed that children between 6- and 12-year-old generally focus on the same local facial features as adults, although no direct comparison was made with adults in this study [21]. Their data showed that six year old children were less accurate in expression recognition and fixated less on the facial images than nine- and twelve-year old children, suggesting that some relationship between eye-movements and development of facial expression recognition skills exists. The similarity in fixation distributions between adults and children may suggest that adults and children recruit crucial information for expression recognition from the same facial features. Behaviourally, expression categorization of children aged ten has indeed been shown to be adult-like for several facial expressions, such as happiness, surprise, disgust and fear, but is still below adult levels of performance for angry and sad faces, with low or medium expression intensity levels [4]. When more difficult discriminations are required, for example in a task using face stimuli morphed from one expression to another, adults show a greater sensitivity in expression recognition than adolescents [4], consistent with the idea that recognition skills still take several years to fully develop after childhood [7]. Based on these behavioural findings, it is reasonable to assume that in terms of recognition accuracy, children are more likely to benefit from facial expression recognition training than adults in the present study. If categorization accuracy improves more in children than in adults after training and behavioural improvements are facilitated by changes in gaze strategy, then spontaneous changes in gaze-patterns can be expected to be more pronounced in children than in adults.

To investigate if training-related changes in performance and gaze-patterns transfer to faces of different people, faces of new models were added in the last training session. In comparison with unfamiliar faces, viewing of personally familiar faces for face identity recognition is associated with more fixations and longer scanning duration [22]–[23], or is accompanied by directing sequential fixations to different local facial regions [24]. Consistent with these findings, training in expression recognition may also result in differential viewing strategies for trained and new faces when categorizing facial expressions. The alternative is that this difference in gaze-strategy for familiar and new faces may not be necessary when facial expression categorization is required.

## Method

### Participants

Sixteen Caucasian adults (8 males and 8 females, mean age = 21.6±3.6) and sixteen Caucasian children (9 boys and 7 girls, aged between 8 years and 2 months and 9 years and 3 months with the mean of 8 years and 8 months±4.5 months) were recruited for this study. All participants had normal or corrected-to-normal visual acuity. Teaching staff was asked to exclude any children with known developmental or visual disorders from participating in the study. Ethical approval was obtained from the Ethics Committee in School of Psychology, University of Lincoln. Written content was obtained from adult participants and written parental consent for the children. Adults were recruited from university student population for course credit and children from a local primary school. Initial pilot study showed that nine-year-old children were able to maintain engagement with the training task in four sessions, yet their performance was still below adult level for some expressions. All procedures complied with the British Psychological Society “Code of Ethics and Conduct” and with the World Medical Association Helsinki Declaration as revised in October 2008.

### Materials and Procedure

Children and adults were trained in four training sessions with a self-paced, free-viewing facial expression categorization task with feedback, using emotional faces with varying levels of expression intensity. Eye-movements were recorded in the first and fourth session to explore training-related changes in gaze-distribution. An additional set of faces was included in the last session to investigate transfer of learning in behavioural and eye-movement measures.

Digitised grey-scale face images in full frontal view were displayed on the monitor of a mobile eye-tracking system (Tobii 1750). Image size was 15.9×12.1° at 70 cm viewing distance. Four western Caucasian faces (two female and two male models) were selected from the Karonlinska Directed Emotional Faces [25]. Each model expressed no emotion (neutral), happiness, fear, or sadness at high intensity. Only three expressions were used to ensure that the training task was not too long for sustained task engagement in children. These expressions were chosen due to reported expression-specific gaze-patterns for sad and happy expressions [20] and established effect of gaze-instructions on fearful expression [15].

The faces were processed in Adobe Photoshop to remove external facial features and to ensure a homogenous grey background, same face size and brightness. For each of the three expressions of each model, Morpheus Photo Morpher was used to create 10 levels of intensity ranging from 10 to 100% with 10% increments by morphing the emotional face with the neutral face Images with intensity levels between 70 and 90% were not included in the experiment. Thus 22 images were created for each model (1 neutral face and 21 expressive faces, 7 per expression), resulting in a total of 88 face images.

Adults and children were tested in a quiet room at the university or at the local primary school, respectively. After practice, participants were instructed to free-view the image and to press a central key when an emotion was recognised, and then to press individual buttons representing individual expressions. This procedure was chosen to reduce variability in response times.

To calibrate eye-movements in sessions 1 and 4, a small red circle (0.4° diameter) was presented randomly at one of nine locations. Each experimental trial started with an animated bee (1.6×1.8°) that moved along a random path within an area of 1.96×2.12° at the screen centre. After 500 ms, the bee was replaced by a face image which remained on the screen until a response was made. Error feedback was provided in the form of a tone (3700 Hz, 7 ms) on trials where participants made an incorrect categorization.

Testing took place in four sessions on four successive days. Participants saw 44 face images of two models in the first three sessions. Forty-four new face images of two new models with the same facial expressions were included in session four. The ‘trained’ and ‘new’ face sets were counterbalanced within each participant age group. Testing sessions 1, 2 and 3 lasted 10–15 minutes per session, session 4 lasted 20–35 minutes.

Eye positions were recorded using a remote eye-tracker system (Tobii 1750) with a 50 Hz sampling frequency and 1° accuracy. The fixations were determined through Tobii Software Development Kit functions with the established Dispersion-Threshold Identification method [26]. Regions of interest (ROI) in face stimuli were eyes (including eyes and eye-brows), nose (including glabella, nasion, tip-defining points, alar sidewall and supra-alar crease) and mouth region. The ROI shape varied slightly across different face models, but the overall area size (20 cm^2^) was the same for all regions in all images. Number of fixations and viewing time allocated to each ROI were normalized to the total number of fixations and total viewing time sampled in one trial. To reduce artefacts of fixations on the bee prior to face presentation, first recorded fixations were excluded in the calculation of total fixation numbers.

## Results

### Effect of training on behavioural measures

To analyse behavioural improvement across the four training sessions, accuracy (percentages of correct expression categorization) and mean Response Times (RT) were entered in a 5 (Training: Session 1, 2, 3, 4-trained faces, and 4-new faces) × 3 (Expression: Fearful, Happy, Sad)×7 (Intensity: 10, 20, 30, 40, 50, 60, 100%)×2 (Age group: Adults, Children) Mixed Repeated Measures ANOVA. Neutral faces were analysed separately with the factors Training and Age group. Greenhouse-Geisser adjustment was applied where appropriate and Bonferroni adjusted t-tests were used for post-hoc analyses. Only significant main and interaction effects were reported and interaction effects with Age-group are reported first.

#### Accuracy

**Figure 1:**
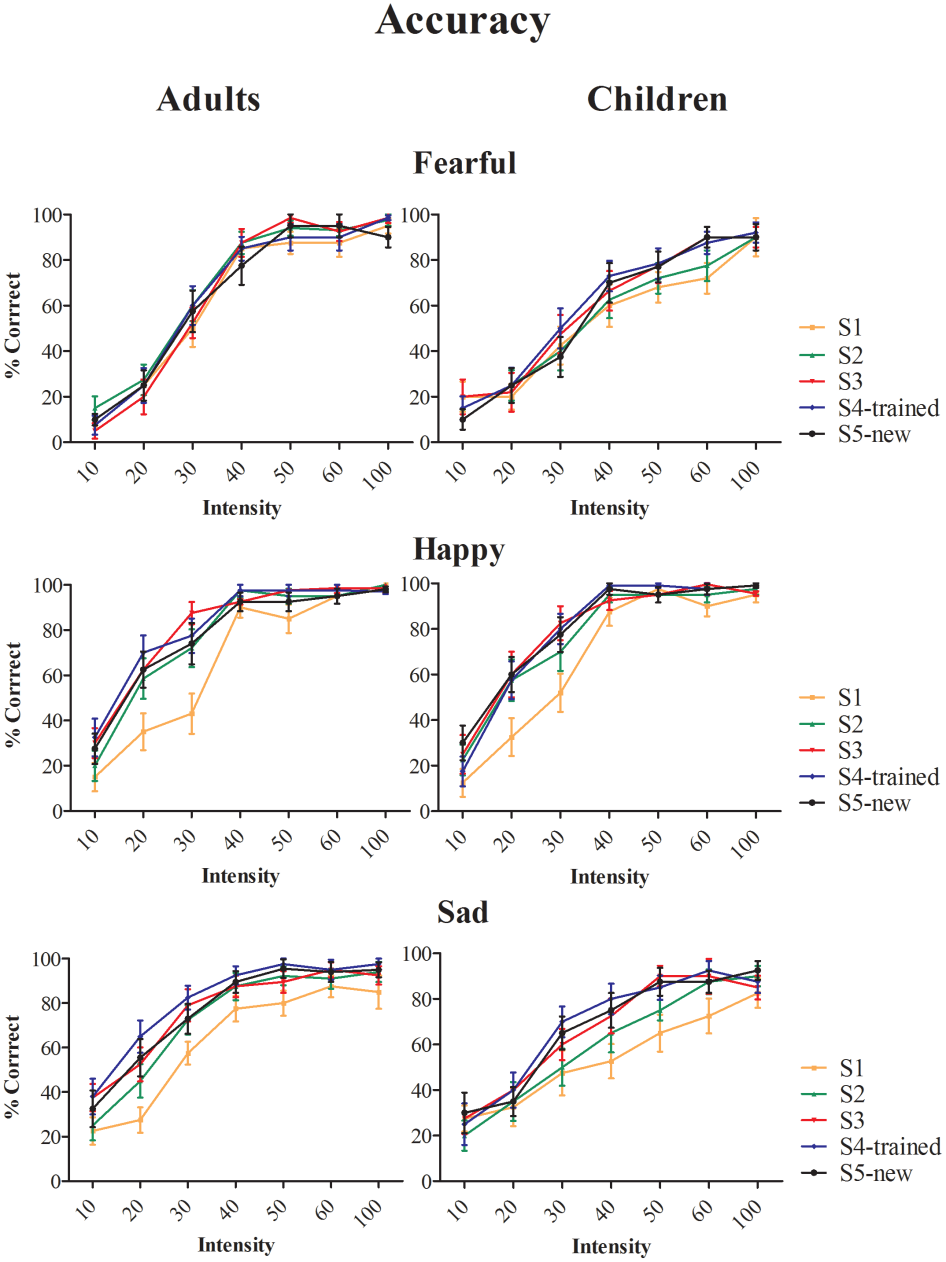
Accuracy: Proportion correct responses (% correct) as a function of Training (S1 = Session 1, S2 = Session 2, S3 = Session 3, S4-trained = Session 4, trained faces, S4-new = Session 4, new faces), Emotion (Fearful, Happy or Sad) and Intensity (0, 10, 20, 30, 40, 50, 60 or 100%) and Age-group (Adults and Children).

Figure 1 shows percentage correct expression categorization responses for adults and children. The analysis of accuracy revealed that overall, the percentage of correct responses was higher for adults than children [F(1,30) = 11.9, *p<0.002, η_p_^2^ = 0.28*], particularly at mid-range intensities for fearful and sad faces [Expression × Intensity × Age-group: F(12,360) = 1.9, *p = 0.03, η_p_^2^ = 0.16*]. Differences between adults and children were significant at 30–50% intensity levels for fearful faces and at 20–50% intensities for sad faces (*all ps≤0.01*). All remaining significant effects were the same for adults and children. Significant effects were found for Training [F(4, 120) = 14.6, *p<0.001, η_p_^2^ = 0.32*], Intensity [F(6,180) = 398.53, *p<0.001, η_p_^2^ = 0.93*], Expression [F(2,60) = 19.8, *p<0.001, η_p_^2^ = 0.39*], Training × Intensity [F(24,720) = 1.59, *p = 0.04, η_p_^2^ = 0.05*] and Training × Expression [F(8,240) = 23.2, *p = 0.002, η_p_^2^ = 0.09*]. Training improved accuracy significantly from session 1 to 2 (*p<0.001*), from session 2 to 3 (*p = 0.01*), and from session 3 to 4-trained faces (*p = 0.02*). Accuracy for new faces in session 4 was significantly better compared to session 2 (*p = 0.03*), but lower than for trained faces in session 4 (*p = 0.04*). Significant improvements across training sessions were only observed for intensity levels between 20% and 60% [F≥3.6, *p≤0.008* for analyses at each intensity level collapsed over Emotion and Age-group]. Figure 1 shows that Training increased accuracy for happy and sad faces, whereas improvements for fearful faces were absent in adults and only minimal for children (improvement in children was only significant at 60% intensity from session 1 to trained and new faces in session 4: *ps≤0.04*).

Analysis of the neutral condition revealed that Training significantly reduced categorization accuracy of neutral faces [F(4,120) = 10.38, *p<0.001*, *η_p_^2^ = 0.28*]. This effect was explained by a significant reduction in accuracy from 60% in session 1 to 30% in session 2 (*p = 0.024*): No further reduction in accuracy was observed after session 2.

#### Response times

**Figure 2:**
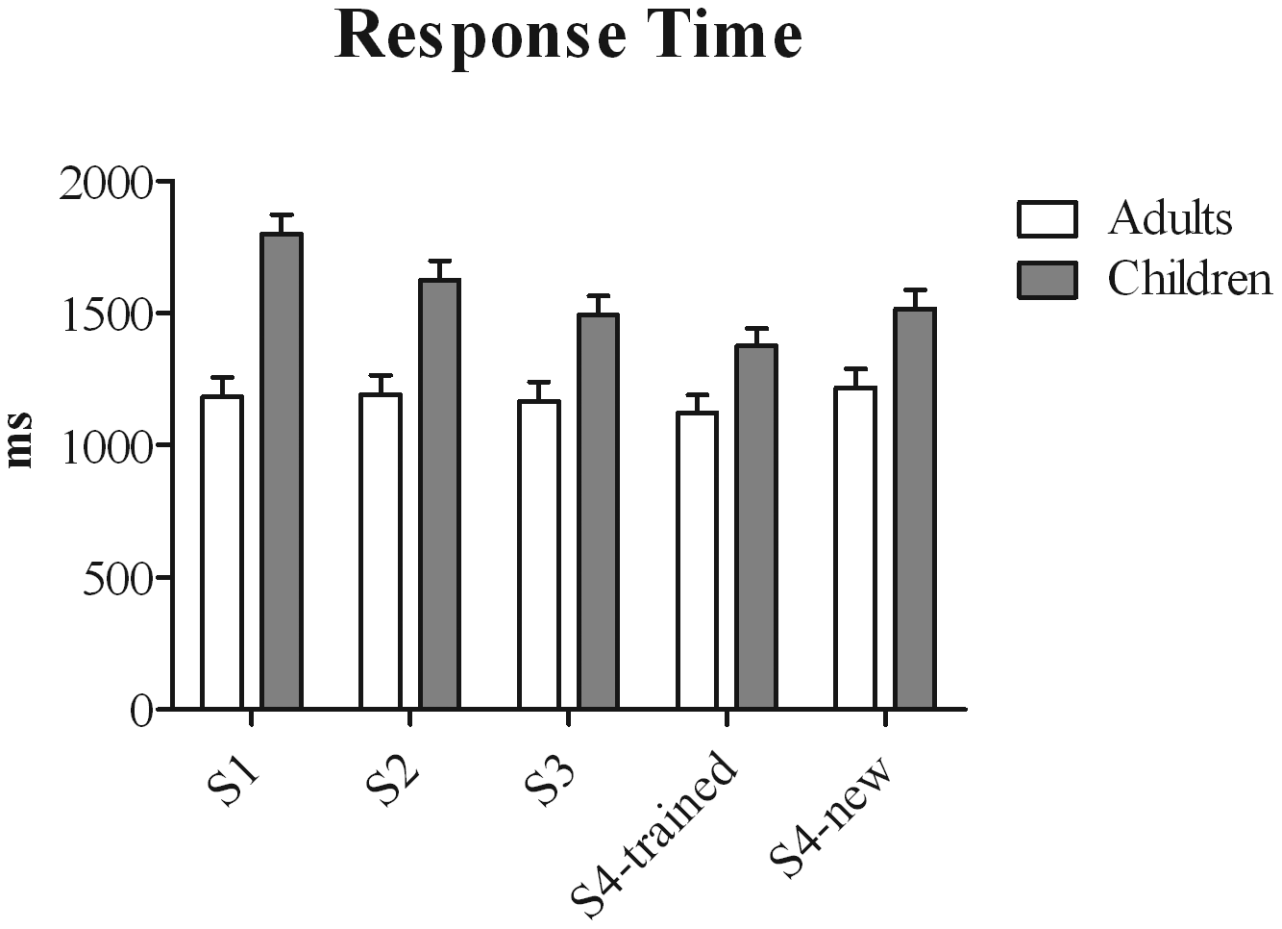
Response Times (RT) in milliseconds (ms) for Adults and children as a function of Training Session.

RT (Figure 2) was slower for children than for adults [Age-group: F(1,30) = 20.68, *p<0.001, η_p_^2^ = 0.41*] and training reduced RT more in children than in adults [Training × Age-group: F(4,120) = 5.83, *p = 0.001, η_p_^2^ = 0.15*]. Separate analysis per age-group showed that the effect of Training was not significant in adults, whereas in children, RT reduced significantly after each training session (*all ps≤0.05*). The remaining significant effects did not interact with Age-group. RT to happy faces was faster than to fearful and sad faces (*ps≤0.001*) [Expression: F(2,60) = 19.3, *p<0.001, η_p_^2^ = 0.39*] and RT decreased gradually as intensity levels increased [F(6,180) = 58.8, *p<0.001, η_p_^2^ = 0.66*].

Analysis of RT for neutral faces only revealed a significant effect of Age-group [F(1,30) = 4.46, *p = 0.43*, *η_p_^2^ = 0.13*] due to shorter overall response times of adults.

#### Incorrect responses

The analysis of accuracy showed that the number of errors made reduced with training. Incorrect responses were analysed further to explore possible response biases. Given the low number of errors made, particularly at higher intensities, non-parametric tests were considered to be most appropriate. Statistical tests of frequency distributions were used (χ^2^ Goodness of Fit and χ^2^ test of associations). Eighty-two percent of all errors recorded were made for face images with a lower expression intensity levels (10–30%), 86% for adults and 78% for children. Overall, low intensity facial expression (10–30%) were most often incorrectly categorised as expressing no emotions (∼50% for all three facial expressions, see Table 1), but for the remaining incorrect categorizations, the type of errors made were different for each emotion. At both low (10–30%) and medium/high (40–100%) intensity levels, fearful faces were more often categorized as sad than happy, whereas sad faces were more often incorrectly identified as fearful than as happy. In contrast, happy faces were equally likely mistaken for sad or fearful faces. The distributions of frequencies across the three emotions differed significantly from chance in all analyses (see Table 1). Further analysis with tests for associations showed that these frequency distributions were not associated with Age-group or Training, suggesting that the type of incorrect categorizations made was similar for adults and children and consistent over the four training sessions for both trained and new faces in session 4.

**Table 1:** Incorrect categorisation responses in percentages as a function of facial expression (fearful, happy, sad) and age-group (adults, children). ‘NE’ = No expression collapsed over Intensity levels (I) 0–30% and 40–100%.

		I = 0–30%		I = 40–100%	
		*Adults*	*Children*	*Adults*	*Children*
**Fearful**	*Happy*	14	13	15	17
	*Sad*	38	41	77	75
	*NE*	48	46	8	8
	*χ^2^*	*18.3*	*18.9*	*86.5*	*79.3*
	*p*	*<.001*	*<.001*	*<.001*	*<.001*
**Happy**	*Fearful*	22	19	45	49
	*Sad*	27	28	38	35
	*NE*	50	53	17	16
	*χ^2^*	*13.5*	*18.6*	*12.7*	*16.4*
	*p*	*0.001*	*<.001*	*0.002*	*<.001*
**Sad**	*Fearful*	31	35	58	61
	*Happy*	19	17	27	22
	*NE*	50	48	15	17
	*χ^2^*	*14.6*	*14.5*	*29.5*	*34.8*
	*p*	*<.001*	*<.001*	*<.001*	*<.001*

Neutral faces were more often mistaken for sad (adults: 49%; children 48%) or fearful faces (adults: 39%; children: 34%) than for happy faces (adults: 12%; children: 14%). No significant associations with Age-group or Training were found for neutral faces.

### Effect of training on Eye-movements

Three different eye-movement measures were analysed: *1*) The total number of fixations made for the duration of face presentation, *2*) The proportion of fixations and viewing times on different facial features for the duration of face presentation and *3*) The proportion of fixations and viewing times of different facial features during the second fixation.

#### Total number of fixations

**Figure 3:**
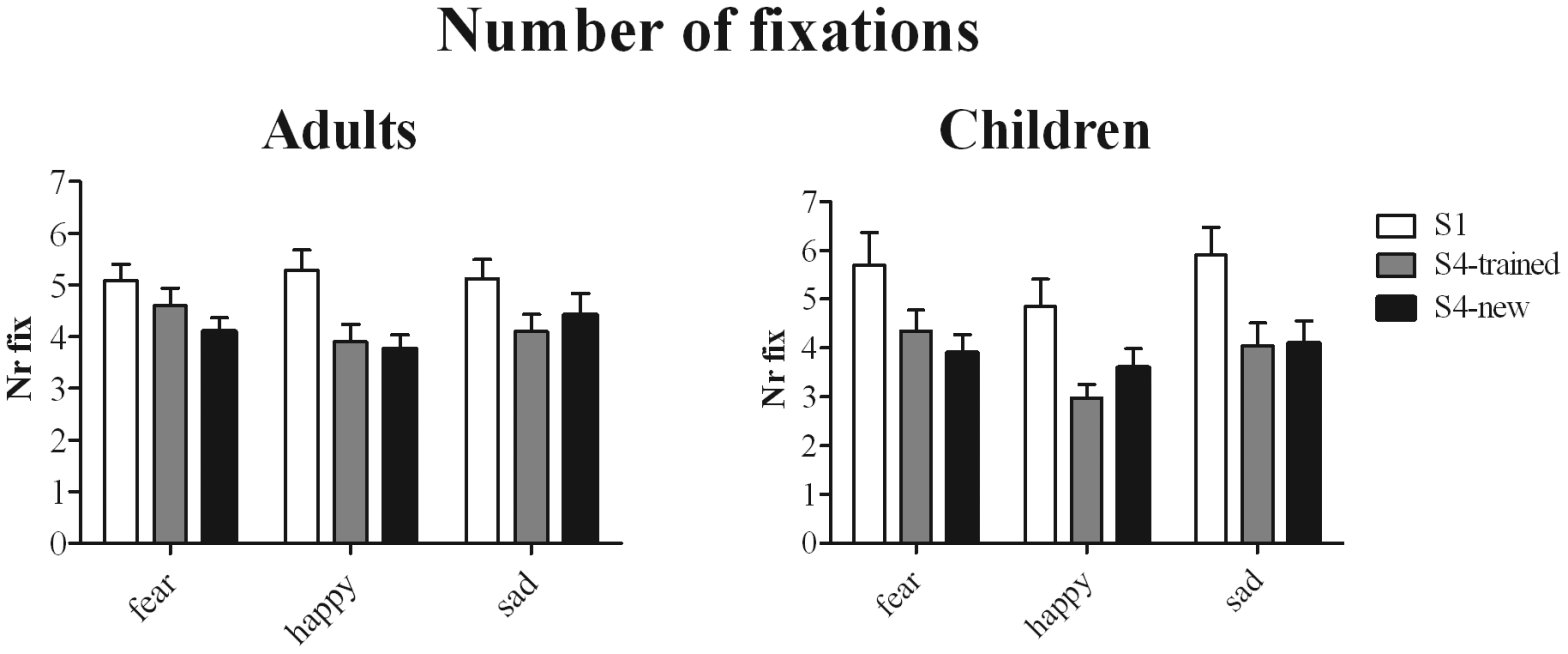
Total number of fixations made on average during face viewing (Nr Fix) as a function of Training and Emotion.

Number of fixations were entered in 3 (Training: sessions 1, 4-trained faces and 4-new faces)×3 (Expression)×7 (Intensity)×2 (Age-group) ANOVA. A significant interaction was found for Age-group × Expression [F(2,60) = 4.4, *p = 0.02, η_p_^2^ = 0.13*]. Figure 3 shows that children fixated more often on sad and fearful faces compared to happy faces (*ps<0.02*), whereas this effect was absent in Adults. All remaining effects were similar for adults and children. Significant effect were found for Training [F(2,60) = 25.6, *p<0.001, η_p_^2^ = 0.46*] and Intensity [F(6,180) = 29.7, *p<0.001, η_p_^2^ = 0.49*]: More fixations were made in session 1 than in session 4 (for all comparisons between session 1 and trained or new faces in session 4: *ps≤0.001*), and the number of fixations made reduced at higher intensity levels. A significant effect of Training × Intensity was found [F(12,360) = 2.14, *p = 0.02, η_p_^2^ = 0.06*]. Further analysis, separately per intensity level, showed that training only reduced fixations for intensity level 20% and higher [F(2,60)≥7.6; *p≤0.001*]. A significant effect was further found for Expression × Training [F(4,120) = 3.8, *p = 0.006; η_p_^2^ = 0.21*]. This interaction effect was best explained by the results of further analysis of Emotion effects, separately for session 1, session 4-trained faces and session 4-new faces. When collapsed over both groups, the difference in the number of fixations for fearful, happy and sad faces was not significant in session 1, whereas for both trained and new faces in session 4, happy faces were fixated less often compared to fearful faces (*ps<0.001*) and sad faces (*ps≤0.02*), suggesting that training reduced fixations more for happy than for sad and fearful faces.

Analysis of neutral faces showed a similar trend: Training reduced the total number of fixations made [F(2,60) = 3.23, *p = 0.046, η_p_^2^ = 0.15*]. Compared to session 1, the number of fixations reduced significantly for both trained faces (*p = 0.04*) and new faces (*p = 0.03*) in session 4.

#### Proportions fixations and viewing times for the whole duration of face viewing

**Figure 4:**
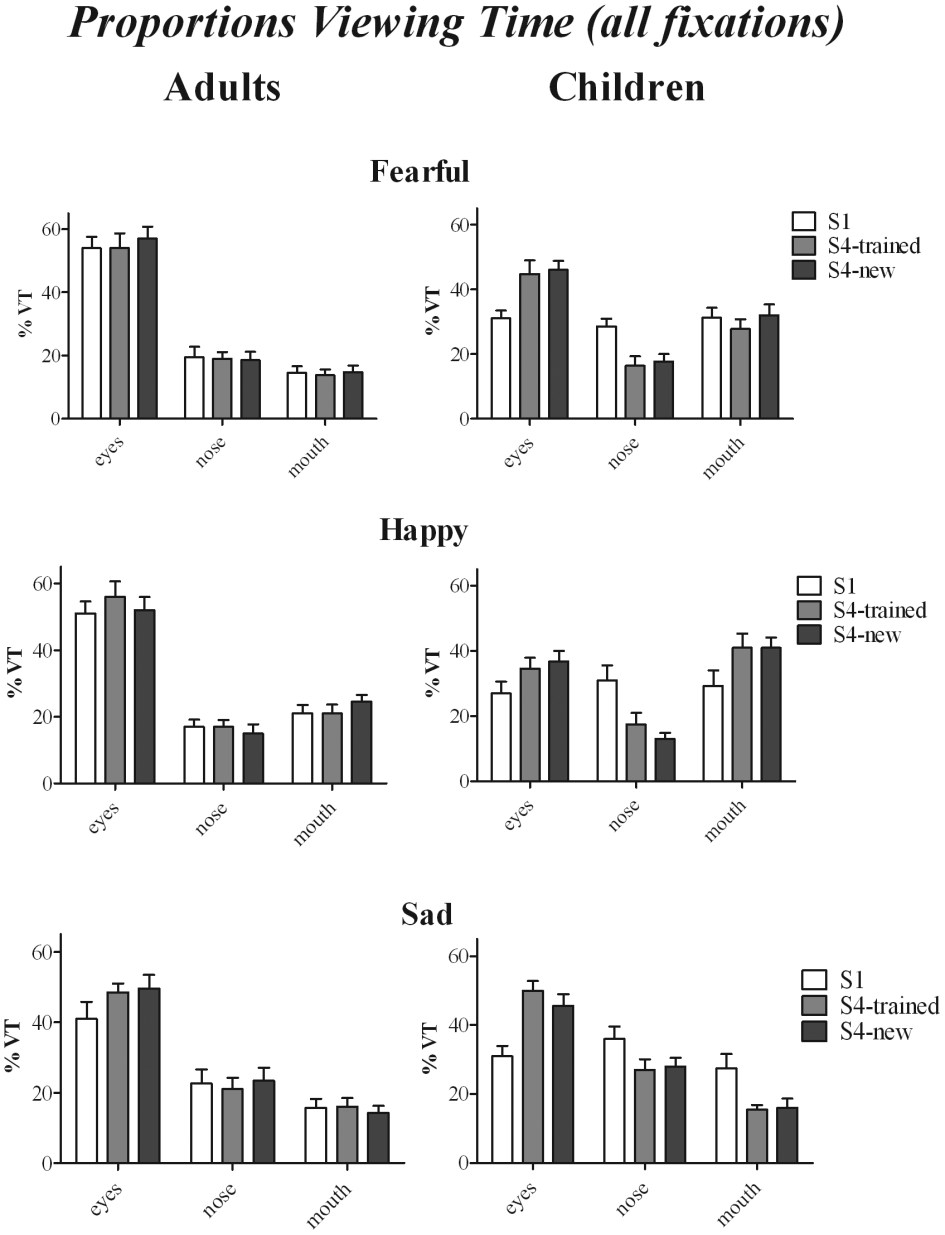
Average Proportions Viewing Time (considering all fixations during face-viewing) as a function of Training session, ROI (Region of Interest), Emotion and Age-group.

The trends and statistical effects for proportion fixations and viewing times (see Figure 4) were similar and are reported together. It will be stated clearly where statistical effects for both measures deviate. Statistical effects that are the same for both measures will be reported first.

On average, 91% of all fixations (92% for adults, 91% for children) and 90% of total viewing time (89% for adults, 91% for children) within a given trial was allocated to one of the three ROI (eyes, nose and mouth). Proportion fixations and viewing times were entered in two separate 3 (Training: sessions 1, 4-trained faces and 4-new faces)×3 (Expression)×7 (Intensity)×3 (ROI: eyes, nose, mouth)×2 (Age-group) ANOVA.

The analyses revealed a significant effect of ROI [Fixations: F(2,60) = 33.1, *p<0.001, η_p_^2^ = 0.51*; Viewing time: F = 45, *p<0.001, η_p_^2^ = 0.6*] and ROI × Age-group [Fixations: F(2,60) = 7.35, *p = 0.002, η_p_^2^ = 0.2*; Viewing time: F = 10.3; p*<0.001, η_p_^2^ = 0.25*]. Both children and adults looked most often and for longest at the eyes (*ps≤0.024*), but compared to adults, children looked less often and for a shorter duration at the eyes (*ps*≤0.002), and more often and for longer at the mouth (*ps≤0.001*). A significant effect for both measures was further found for ROI x Expression [Fixations: F(4,120) = 7.34, *p<0.001, η_p_^2^ = 0.2*; Viewing time: F = 22.5, *p = 0.001, η_p_^2^ = 0.42*] which did not interact significantly with Age-group. Overall (when measures were collapsed over both age-groups) the nose was fixated for longer when viewing sad faces compared to happy or fearful faces (*ps≤0.012*) and the mouth when viewing happy faces compared to sad and fearful faces (*ps≤0.018*). Importantly, a significant effect was found of ROI × Training × Age group [Fixations: F(4,120) = 4.56, *p = 0.002, η_p_^2^ = 0.13*; Viewing time: F = 3.9, p* = 0.05, η_p_^2^ = 0.12*]. Figure 4 shows that training increased proportion fixations and viewing times of the eyes in both adults and children. For adults however, this effect was only significant for sad faces for both measures (session 1 vs. trained or new faces in session 4: *ps≤0.05*) whereas for children, increased viewing of the eyes (and reduced viewing of the nose) was significant for all three expressions (for all comparisons between session 1 and session 4-trained and new faces: *ps≤0.03*). *In viewing time only*, significant effects were also found for ROI × Training × Expression [F(8,240) = 3.5, *p = 0.001, η_p_^2^ = 0.1*] and ROI × Training × Expression × Age-group [F(8,240) = 1.96, *p = 0.05, η_p_^2^ = 0.06*]. Figure 4 illustrates that only children showed expression-specific training effects in viewing time, reflected in longer viewing of the mouth for trained and new happy faces in session 4 compared to session 1 (*ps≤0.002*), and reduced viewing of the mouth for trained and new sad faces after training (*ps≤0.02*). Comparisons between adults and children further showed that in session 1, for all three expressions, adults looked longer at the eyes compared to children (*p≤0.04*) and shorter at the nose (*ps≤0.04*) and the mouth (*ps≤0.03*). After training, the viewing time distribution for sad faces (trained or new) was similar for adults and children, whereas for happy and fearful faces, adults still looked longer at the eyes compared to children (trained or new: *ps≤0.02*), and children looked longer at the mouth compared to adults (*ps≤0.001*).

Effects of training on eye-movements for neutral faces followed a similar trend. For both measures, significant effects were found for ROI x Group [Fixations: F(2,60) = 6.67, *p = 0.002, η_p_^2^ = 0.18*, Viewing times: F = 7.9, *p = 0.001, η_p_^2^ = 0.18*] *and* Training x ROI [Fixations: F(4,120) = 2.85, *p = 0.027, η_p_^2^ = 0.09*; Viewing times: F = 3.38; *p = 0.012, η_p_^2^ = 0.10*]. Compared to adults, children looked more often and for longer at mouth (*ps≤0.005*) and less often and for a shorter duration at the eyes (*ps≤0.001*). Overall (when proportions were collapsed over age-group), training increased viewing of the eyes for both trained and new neutral faces (*ps≤0.001*).

#### Proportion fixations and viewing times at early stages of face viewing − second fixation

**Figure 5:**
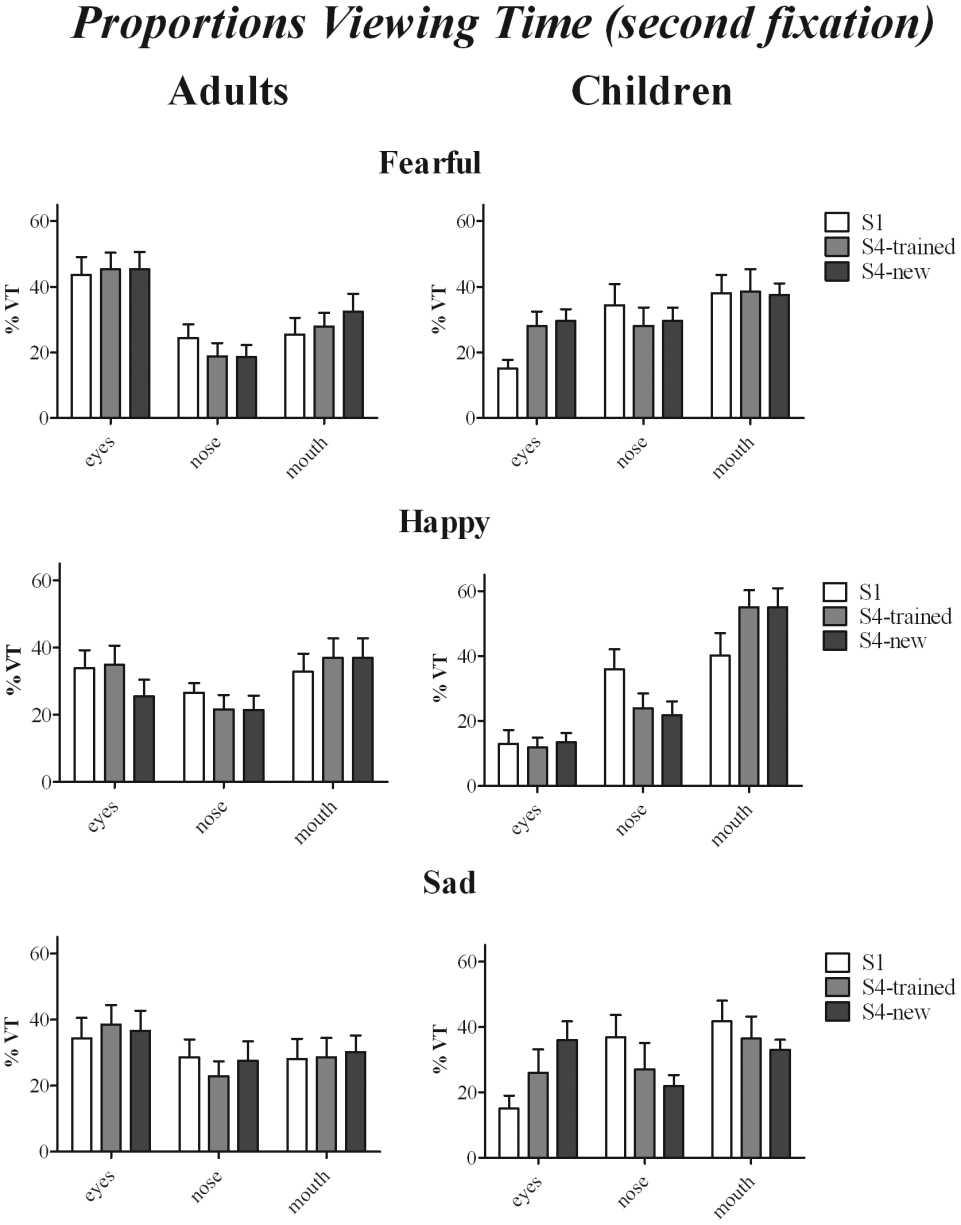
Average Proportions Viewing Time (second fixation only) as a function of Training session, ROI, Emotion and Age-group.

To obtain a measure for analysis of gaze distribution at early stages of face viewing, proportion fixations and viewing times during the second fixation were collapsed over intensity level (See Figure 5). These average proportions fixations and viewing times were entered in two separate 3 (Training: sessions 1, 4-trained faces and 4-new faces)×3 (Expression)×3 (ROI: eyes, nose, mouth)×2 (Age-group) ANOVAs. The third and fourth fixation were not analysed given the low number of fixations made for facial expressions at 100% intensity after training (particularly for happy faces, see Figure 3). Analysis of second fixations revealed a significant effect of ROI × Age group [Fixations: F(2,60) = 4.23, *p = 0.019, η_p_^2^ = 0.12*; Viewing time: F = 4.7, *p<0.03, η_p_^2^ = 0.13*]: Whilst adults looked more often and for longer at the eyes compared to the nose (*ps≤0.05*), children viewed the mouth more often and for longer compared to the eyes (*ps≤0.024*). Significant effects were further found for ROI×Training [Fixations: F(4,120) = 3.73, *p = 0.007, η_p_^2^ = 0.11*; Viewing time: F = 2.8, *p<0.028, η_p_^2^ = 0.09*], and *in viewing time only*, for ROI × Training × Expression [F(8,240) = 2.96, *p = 0.003, η_p_^2^ = 0.09*] and ROI × Training × Expression × Age-group [F(8,240) = 2.25, *p = 0.03, η_p_^2^ = 0.07*]. Further analysis, separately for each age-group, revealed that the interaction effects for Training × ROI and Training × Expression × ROI were not significant in adults, suggesting that training had no significant effect on allocation of the second fixation in adults. Figure 5 shows that the effects of training in children were expression-specific: After training children looked longer at the eyes in fearful and sad faces, but looked longer at the mouth in happy faces (session 1 vs. session 4-trained or new faces: *all ps≤0.04*).

### Further analysis

#### Training effects in session 1

The design of this study did not include a pre-training assessment of baseline performance. We therefore compared behavioural and eye-movement measures for first and second half (‘Block’) of the first session to investigate whether the effect of feedback changed performance significantly within this session. For behavioural measures and for the total number of fixations made, the effect of Block was analysed separately for the factors Intensity and Expression. To analyse whether gaze distribution changed in the first and second block, proportion fixations and viewing times were entered in ANOVAs with the factors Block, ROI and Expression.

##### Behavioural measures

No effect of Block was found for Accuracy (Block: F(1,30) = 2.4, *p = 0.12, η_p_^2^ = 0.08*; Block × Age-group F(1,30) = 1.07, *p = 0.74*, *η_p_^2^ = 0.004*]. For RT, borderline significant effects were found for Block [F(1,30) = 4.1, *p = 0.05, η_p_^2^ = 0.08*] and for Block × Age group [F(1,30) = 3.9, *p = 0.06, η_p_^2^ = 0.09*]. On average, RT reduced more for children (from 1848 to 1752 ms) than for adults (from 1202 to 1172 ms) from the first to the second half in session 1. All remaining interaction effects with Block in the analysis of Accuracy and RT were not significant [F≤2.28, *p≥0.14, η_p_^2^≤0.07*].

##### Eye-movement measures

For the total number of fixations made, no significant effects of Block were found [Block: F(1,30) = 2.3, *p = 0.14, η_p_^2^ = 0.07*; Block × Age group: F(1,30) = 0.13, *p = 0.89, η_p_^2^ = 0.009*, all remaining interaction effects: F≤1.49, *p ≥0.23, η_p_^2^≤ 0.05*]. The analysis of proportional fixations and viewing times showed that gaze-distributions did not change significantly from the first to the second half of the first training session [Block × ROI: F(2.60) = 0.31, p = 0.73, *η_p_^2^ = 0.01*, Block × ROI × Age group: F≤1.56, *p ≥0.19, η_p_^2^≤0.05*]. All higher-order interaction effects with the factor Block were also not significant [F≤1.7, *p≥0.15, η_p_^2^≤0.05*].

### Correlations

Correlation analysis was used to explore if training-related changes in behavioural measures were linearly related to changes in gaze-distribution, most characterized by an enhanced focus on the eyes. Accuracy and RT were averaged for the low/mid intensity levels (10–40%, where improvement was most pronounced) and difference values were calculated by subtracting values for session 1 from values in session 4 (‘d-acc’ and ‘d-RT’), separately for trained and new faces. These values were correlated with difference values for proportion of fixations (‘d-fix’) and viewing times (‘d-dur’) towards the eyes (session 4 – session 1). The results of this analyses revealed borderline significant relationships between viewing time of the eyes and RT, for children only [trained faces: *r = 0.48, p = 0.057*, new faces: *r = 0.51, p = 0.049*]. This trend indicates that the more viewing times of the eyes was prolonged with training, the more RT reduced.

## Discussion

The present study revealed that training-related improvements in facial expression categorization (mostly associated with mid-range expression intensity levels) coincided with changes in gaze distribution in face exploration for all three expressions in children and for sad expressions in adults, supporting the assumption that enhancements in expression recognition is facilitated by spontaneous changes in gaze-strategy. Children's gaze behaviour was characterized by a pronounced shift in focus towards the eyes after training, resulting in more adult-like gaze distributions. Importantly, this focus was not influenced by expression intensity at any stage of training, consistent with previous findings [19], or by the introduction of new faces in session four, suggesting transfer-effects of learning.

Guo [19] originally suggested that the insensitivity of gaze distribution to expression intensity reflects the use of a ‘holistic’, uniform viewing strategy to extract relevant facial cues from all internal features when categorizing subtle expressions. The enhanced eye-focus after training in children clearly demonstrates that training increases the relative importance of information within the eye-region, yet it is less clear whether this information benefits holistic or non-holistic (feature-based) processing in children. Holistic viewing generally refers to the ability to simultaneously process multiple cues from the whole face [27]. Developmental studies have demonstrated that adults' expertise in face identity recognition depends at least partly on the ability to process faces holistically, reflected in a shift from analytic, feature-by-feature processing to holistic, configural processing of faces (e.g. processing of spatial relations between facial features) during early childhood, e.g. [28]–[31]. Configural processing has also been shown to be important in face processing for expression recognition in both adults [32]–[33] and children [3]. This can be demonstrated with the composite effect, which refers to reduced recognition accuracy when two face halves, each expressing a different emotion, are aligned (forming the illusion of one face and therefore more likely to elicit configural processing) compared to when face-halves are not aligned. Using these composite faces, Durand et al [3] showed that face-half alignment reduced expression recognition measures to the same extent in children aged between five and twelve, suggesting that holistic processing is used for facial expression recognition from a very young age. Based on these findings, it is plausible to assume that the additional information extracted from the eye-region after training by children includes cues that benefit holistic processing.

At early stages of face viewing (i.e. at the stage when the second fixation is made), adult eye-movement patterns were unaffected by training and their gaze distributions indicate a strong focus on the eye-regions from the beginning of the training. In contrast, children tended to focus more on the nose and mouth in session one for all three expressions and training resulted in emotion-specific gaze-patterns, characterized by increased viewing times of the eyes for fearful and sad faces and on the mouth for happy faces. One possible explanation for this effect may be that at early stages of face-processing, feature-based analysis was enhanced more in children than in adults. The importance of feature analysis in expression recognition has been demonstrated in studies using ‘bubbles technique’ (where only one feature is presented) or feature masking. These studies have shown that observers can recognize high-intensity expressions from specific facial features only, such as the eyes in sad and fearful faces and the mouth in happy faces [34]. On the other hand, a strong reliance on analytic, feature-based processing (as demonstrated by a reduced composite face effect for example) has also been associated with reduced expertise in face processing, such as in patients with congenital prosopagnosia [35]. Over-reliance on facial features in propsagnosic patients has further been found to be associated with a stronger gaze focus on the lower half of the face, including the mouth and nose [36]. Furthermore, whilst previous studies have clearly evidenced that children process faces holistically from a very young age for facial expression recognition [3], other findings suggest that more difficult configural face processing skills, such as processing of small changes in the spatial relations between facial features, develop gradually in later childhood [31]. It will require further studies to investigate to which extent training enhanced feature-based and/or configural processing in children and whether the same processing skills were trained in both age groups. The observation that training enhanced expression-specific gaze-distribution in children only may suggest however, that training increases feature-based processing more in children than in adults, at least at early stages of face-viewing for facial expression recognition.

The observation that children generally focused more on the mouth compared to adults, particularly for fearful and happy faces, was an unpredicted finding. One possible explanation for this mouth-focus could be the relative inexperience of children with the variability in facial muscle movements for each expression [37]. Children are better at discriminating high intensity expressions (e.g., [4]) for which the mouth provides important diagnostic information, such as the open mouth for fearful faces (feargasp), the downward bottom lip for sad faces and the smile for happy faces. It may require more experience and conceptual understanding of emotions to learn that expressions of emotions such as fear and sadness are not always associated with changes in the mouth (e.g., [5]), especially when these facial affects are expressed at low intensities.

Training improved behavioural performance beyond the trained face-set, demonstrating transfer-effects of learning. Our eye-movement data further showed that gaze-distributions were almost identical for trained and new faces in session four. This remarkable similarity raises questions about the nature of the learning processes underlying the training-related changes. One possible explanation is that the changes in gaze-strategy reflect a learning strategy that benefits face processing more generally: In addition to facial expression categorization, a stronger eye-bias may also be advantageous for extraction of crucial cues for identity judgements. Consistent with this idea, Heisz and Shore [23] showed that training face identity recall over four successive days resulted in a stronger eye-focus in face viewing as familiarity with the faces increased. A related question is whether perceptual learning effects may have influenced face processing in general due to repeated exposure with face stimuli. Effects of mere exposure have been demonstrated in several studies for face identity discriminations [38]–[40]. Heron-Delaney et al [39] showed for example that the development of ‘own-race effect’ (characterized by enhanced identity discrimination of members of one's own race) in Caucasian infants can be eliminated by exposing children to booklets containing a variety of faces of a different race between the age of 6 and 9 months. Interestingly, discrimination at 9 months was assessed using a new face set, suggesting that perceptual learning due to exposure transferred to new identities. A recent study further showed that 9 month old infants have a stronger eye-focus when viewing own-race compared to other-race faces, suggesting that enhanced viewing of the eyes may be associated with exposure and experience at a very young age [41]. These questions about the relative contribution of general (perceptual) learning effects in training facial expression categorization could be investigated further by directly comparing the benefits of supervised and unsupervised learning in facial expression and identity recognition training tasks.

There are a few methodological issues that will require further investigation. Firstly, the effect of training may have been slightly underestimated by the absence of a pre-training baseline performance assessment, particularly for adults. Whilst inclusion of this condition would have improved the design, analysis of session one revealed no significant differences between first and second session half (other than a small overall reduction in response times in the second half for children) and no interaction effects with age group, suggesting that training effects in session one were similar for both age groups.

Secondly, effect size of higher order statistical effects were generally small, most likely due to relatively small sample size. Inferences based on these results should therefore be considered with some caution and will require verification in future studies. This issue also applies to the borderline significant relationship found between training-related reductions in response times and increased viewing of the eyes for children, which could suggest that a stronger focus on the eyes reduced the time required for recognition. A replication of this result will be necessary to confirm this finding.

Third, the benefits of training on accuracy were only marginally greater in children than in adults and restricted to a few mid-range intensities for sad and fearful faces. This may suggest that the relationship between behavioural improvements and changes in gaze-distributions observed in our study may not be as linear as originally predicted. However, at this stage it is not clear to which extent this finding could be explained by ceiling effects in performance or whether more sessions are perhaps required to improve recognition accuracy of children, particularly for fearful and sad faces. The observation that performance for fearful faces improved for children but not adults could indicate that children may benefit more from additional training sessions.

Fourth, whilst training increased accuracy for low and mid-range intensities, neutral faces were more often incorrectly categorized after training. One plausible explanation may be the relative low probability of trials requiring a ‘no expression’ response (∼4.5%) compared to fearful, sad or happy responses (∼31.8% for each expression) used in the present task, which may have resulted in a bias towards categorizing a face as ‘emotional’ after training. If this explanation is correct, then including more neutral faces should reduce this effect.

The findings of the present study raise several questions for future studies. Firstly, as yet it is not clear to which extent the effect of training on gaze-strategy is influenced by the range of expression intensities used. Whilst a general bias towards the eyes may be most beneficial when a large proportion of face stimuli express emotions at low intensities, gaze-patterns may become more emotion-specific after training when mostly high intensity expressions are included, where specific facial features may become more diagnostic for discriminating between different facial expressions.

The observation that training effects transferred to new faces raises questions about the extent of these transfer effect. For example, further investigations are needed to explore whether training on one set of facial expressions will transfer to a new set of emotional expressions. In addition, based on previous findings demonstrating cultural diversity in the importance of different facial features for expression recognition [42], further studies are required to explore if transfer-effects may be influenced by cultural differences, for example by including new faces of a different cultural group in the last training session. Furthermore, to establish the efficacy of the training task, it will be important to investigate whether training benefits in terms of categorization accuracy and gaze-strategy coincide with improvements in other measures of social functioning in children.

An additional outstanding question concerns the stages of stimulus processing that are most affected by training. Psychophysical studies have found that the benefits of visual perceptual learning can be limited to the trained stimulus property (e.g. a specific orientation or location), indicating the involvement of low-level visual processes in learning (e.g., [43]). Our observation of improved expression categorization to the untrained faces suggests that learning in our task was not just restricted to early visual processing and may have influenced processing at several stages between stimulus onset and response execution. We are currently investigating this question by recording Event-Related Potentials before and after training.
